# Transient effects of carotid baroreflex stimulation via the neck chamber device on central venous pressure

**DOI:** 10.1111/jch.14387

**Published:** 2021-11-16

**Authors:** Fosca Quarti‐Trevano, Gino Seravalle, Domenico Spaziani, Jennifer Vanoli, Giuseppe Mancia, Guido Grassi

**Affiliations:** ^1^ Clinica Medica Department of Medicine and Surgery University of Milano‐Bicocca Milan Italy; ^2^ Unità Operativa Complessa di Cardiologia Unità di Elettrostimolazione Magenta Hospital Magenta Milan Italy; ^3^ Policlinico di Monza Monza University Milano‐Bicocca Milan Italy

## Abstract

We examined in 11 young subjects (age 29.7±3.6 years, mean±SEM) whether carotid baroreceptor stimulation via the neck chamber device may affect central venous pressure (CVP), thus potentially involving other reflexogenic areas in the examined responses. Application of progressively greater neck chamber subatmospheric pressures caused a progressive lengthening in RR interval, which reached a peak at the maximal value of negative neck chamber pressure applied. This was accompanied by significant and progressively greater reduction in CVP values when the data were calculated considering the early changes occurring within the first 2 seconds of the stimulus. There was a weak correlation between the early changes in CVP and the RR interval responses when all stimuli were pooled together (r = 0.32, *P* < .05). The results of the present study suggest that the neck chamber technique employed to assess carotid baroreceptor‐heart rate sensitivity can transiently affect via the CVP reduction cardiopulmonary receptors activity, which may participate at the integrated reflex responses.

## 1 INTRODUCTION

Among the various techniques allowing to assess in humans carotid baroreptor modulation of sinus node activity, the approach based on the evaluation of baroreflex control of heart rate (HR) has been and it still remains one of the most common. The approach is based on application of different degrees of subathmospheric pressure around the neck within a neck chamber device allowing to increase carotid baroreceptor activity inducing a reflex bradycardic response.^1^ The technique is employed in clinical studies given its non‐invasivity, its ability to examine a wide range of the stimulus‐response curve as well as its possibility to be used under different experimental conditions.^1^, ^2^ These advantages are counterbalanced by clearcut limitations, such as the variability of the HR responses within a given patient when the stimulus is repeated different times leading to a reduced reproducibility of the responses examined.^3^ Additional limitations include (1) the fact that the HR responses are potentially affected by different breathing phases^4^ and (2) the exact extent to which pressures are transmitted to the carotid sinuses is difficult to be precisely quantified in different subjects,^3^ making comparison of the reflex responses between individuals to be performed with caution. Whether and to what extent the approach selectively engages carotid baroreceptors or it also concomitantly involves other reflexogenic areas, as it has been reported for another approach used to test the arterial baroreflex based on the systemic infusion of vasoactive drugs,^5^ is unknown. The hypothesis tested in the present study is that the neck chamber technique may alter, for anatomical reasons, central venous pressure and thus the activity of receptors located within the thickness of the cardiac chambers and known to be sensitive in their in activity to changes in central blood volume.

## 2 MATERIALS AND METHODS

### 2.1 Study population

We evaluated 11 subjects of both sexes (eight men and three women) whose age was 29.7±3.6 years (mean±SEM), while sphygmomanometric blood pressure, HR, and body mass index amounted to 125/78 mmHg, 74.3±3.4 beats/min, and 24.4±0.9 kg/m^2^, respectively. The subjects belonged to a group of individuals referred to the hospital for the occurrence of a first episode of syncope and underwent to a number of diagnostic examinations for testing autonomic responses including carotid baroreceptor stimulation. This was performed after positioning different measuring devices including an EKG, a pneumotacograph and a central venous pressure (CVP) line. Based on the normal responses to current autonomic tests, including the carotid baroreceptor stimulation, in these 11 patients the diagnosis of neurogenic reflex syncope was ruled out. All other clinical conditions potentially affecting autonomic cardiovascular modulation, including heart failure, hypertension, previous myocardial infarction, cardiac arrhythmias, renal failure, and diabetes mellitus were also excluded by anamnestic or instrumental evaluation.^6^ Echocardiographic measurements were all normal. All subjects were in sinus rhythm and none was under any cardiovascular drug treatment. They were selected on the basis of the above‐mentioned criteria and in addition on the agreement to participate at the study. All gave their written informed consent to the study, the protocol of which was approved by the Ethics Committee of the two participating hospital institutions, which adopted the same recruiting and operating criteria for the present study.

### 2.2 Study variables

In each subject, the carotid baroreceptor‐HR reflex was evaluated via the above‐mentioned neck chamber technique.^1^ Subatmospheric pressure within the chamber was applied in four separate steps of 10 seconds duration ranging from ‐7 to ‐ 40 mmHg. Each step was repeated three times to increase the reproducibility of the reflex responses and average responses were calculated for each parameter examined. Steps were applied in a random order and separate from each other by a 2‐minute interval. Before and during each stimulus, the pressure within the chamber was measured by a transducer, whereas HR was measured as RR interval using a standard EKG lead. CVP measurements were performed by a catheter placed in the right atrium from an antecubital vein of an arm and connected with a transducer. The position of the catheter in the right atrium was checked by chest radiography and assessment of the physiological CVP waveforms. Baseline values were obtained by averaging values recorded in the 3 minutes before the stimuli, which were delivered to the patient at the end‐expiration breath hold phase verified by pneumotacographic breathing tracing. The HR and CVP responses to neck pressure application were calculated by considering (1) the maximal lengthening of the RR interval over the three beats following the onset of the stimulus^3^ and (2) the early (occurring within 1 second of the stimulus applied) and the late (occurring in the remaining 8 seconds) changes in CVP. Data from the three stimuli of identical magnitude were averaged and calculations were performed for the three steps of negative pressure applied. Neck pressures, CVP, and EKG recordings were digitized with a sampling frequency of 1000 Hz (Powerlab Recording System Model ML870 8/30, AD instruments, Australia).

### 2.3 Statistical analysis

Data were calculated by an investigator unaware of the experimental design. Values from individual subjects obtained before and during carotid baroreceptor stimulation were separately averaged and differences in the values between baseline and the stimulus assessed by two‐way analysis of variance for repeated measurements (ANOVA). Student's t test for paired observations was used to locate the statistical significance of the difference, after the Bonferroni correction. The Pearson correlation coefficient was used to determine the relationships between CVP and RR interval changes. Reproducibility of the CVP and R‐R interval responses to the same level of neck pressure applied within the same session was tested by Pearson's correlation coefficient and Bland‐Altman plot. All analyses were performed with SAS software version 9.3 (SAS Institute Inc, USA). Data are shown as means ± SEM. A *P* vaòue < .05 was taken as the level of statistical significance.

## 3 RESULTS

**FIGURE 1:**
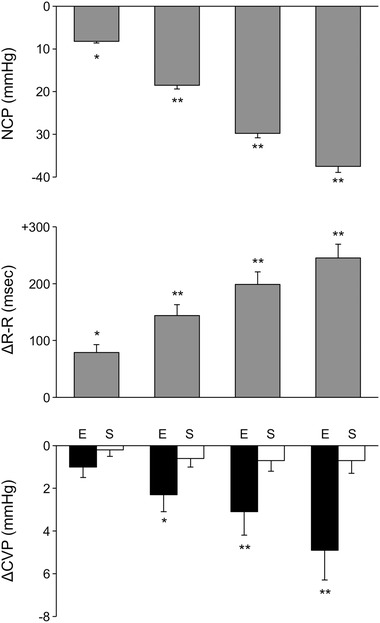
Bar graphs showing the four progressively greater neck chamber pressures (NCP, upper panel), the corresponding lengthenings in the RR interval (middle panel) and the corresponding reductions in central venous pressure (CVP) measured within the first 2 seconds (E) and during the remaining 8 seconds (S) of the stimulus applied. Asterisks (*P < .05, **P < .01) refer to the level of statistical significance of the changes observed in the various variables vs pre‐stimulus control value. Data are shown as means±SEM

**FIGURE 2:**
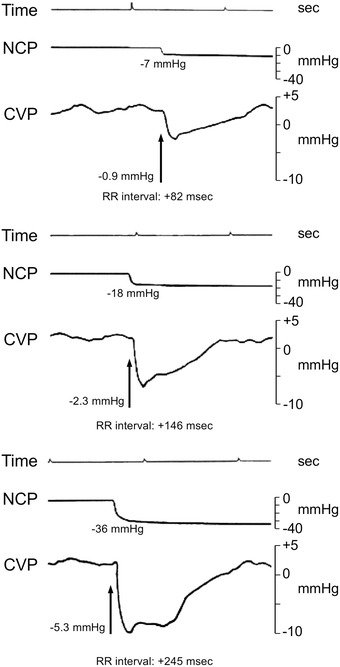
Original tracings of neck chamber pressure (NCP) and central venous pressure (CVP), together with time indication (seconds, sec) and changes in RR interval (msec) in one individual underwent three different neck chamber stimuli. Note the transient reductions in CVP of progressively greater magnitude from top to bottom according to the greater NCP applied

As expected, application of progressively greater neck chamber subatmospheric pressures caused a progressive lengthening in RR interval, which reached a peak at the maximal value of negative neck chamber pressure applied (Figure 1, upper and middle panels). This was accompanied by significant and progressively greater reduction in CVP values when the data were calculated considering the early changes occurring within the first 2 seconds of the stimulus applied (see original recordings in Figure 2 and average values in the lower panel of Figure 1). In contrast, the late CVP changes detected in the remaining 8 seconds of the stimulus were not significant and values were almost superimposable to the ones seen in the pre‐stimulus control period. Bland‐Altman plots showed that in the total population sample the mean difference between the 3 measurements of CVP changes obtained for the same degree of neck chamber stimulus was small (on average 0.3 mmHg), in contrast to the greater differences seen for R‐R interval changes (on average 32 msec).There was a significant, although weak, correlation between the early changes in CVP and the RR interval responses when all stimuli were pooled together (r = 0.32, *P* < .05). No correlation was found when late CVP changes were examined.

## 4 DISCUSSION

The main finding of the present pilot study is represented by the evidence that the neck chamber device used to assess carotid baroreceptor control of HR induced a marked and significant reduction in CVP values. Since CVP changes represent the stimuli for cardiopulmonary receptor activity, the alterations we observed can be taken as markers of an engagement of volume sensitive rectors located within the cardiac walls to the stimulus applied. They can be regarded as an indication that the observed RR interval changes induced by the neck chamber device can depend not only on a carotid baroreceptor stimulation but also on a cardiopulmonary receptor deactivation. Whether cardiopulmonary receptors interfere with the sinus node influences of the carotid baroreceptors is debated.^7^, ^8^, ^9^ It should be emphasized, however, that the interactions between the two reflexogenic areas, if present, should be limited to the very early time of the delivered stimulus, the CVP changes we documented almost completely vanishing after the first 2 seconds of the stimulus applied. This time period, however, immediately preceded the one characterized by the occurrence of the reflex RR responses.

## 5 STUDY LIMITATIONS

The present pilot study has two limitations. First, the population sample of the study was small, preventing definite conclusions to be drawn. Second, we examined patients with a first episode of syncope and, although the neurogenic nature of this episode was excluded, the present data cannot be safely extrapolated to hypertensive without the presence in their clinical history of a single episode of syncope.

## 6 CONCLUSIONS

Data collected in the present pilot study suggest that the neck chamber technique employed to assess carotid baroreceptor‐HR sensitivity can transiently engage cardiopulmonary receptor activity, which may participate at the integrated reflex responses. A similar engagement has been reported also for the arterial baroreceptor testing obtained via the vasoactive drug infusion technique, which has been shown to trigger a much prolonged increase (phenylphrine) or decrease (nitroprusside) in CVP.^5^ Further studies are needed to determine whether and to what extent the observed CVP changes are different as far as time‐course and/or magnitude in patients with cardiovascular disease, in which the carotid baroreflex assessment is quite frequently performed for prognostic and therapeutic evaluation.^6^, ^10^

## CONFLICT OF INTEREST

The authors declare that they have no conflict of interest.

## AUTHOR CONTRIBUTIONS

Dr Spaziani, Seravalle, and Vanoli collected the data, Dr Quarti‐Trevano blindly analyzed them, and Professor Grassi and Mancia wrote the first draft and the revised version of the paper, after comments and criticism by coauthors.
